# Supplementary material for: A simple, single-tube overlapping amplicon-targeted Illumina sequencing assay
Source: PLoS One. 2023 Sep 14;18(9):e0288687. doi: 10.1371/journal.pone.0288687 (PMC10501585; doi:10.1371/journal.pone.0288687)
Supplement: S3 File — (PDF) [file pone.0288687.s003.pdf]

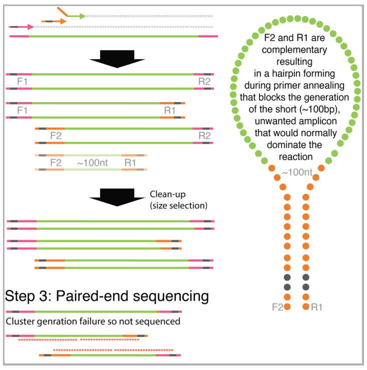

hisPCR: A simple, single-tube overlapping amplicon-targeted Illumina sequencing assay

Jason D Limberis[1], alina.levin[1], Joel Ernst[1], john.metcalfe[1]

[1]University of California, San Francisco

Jason D Limberis
University of California, San Francisco

ABSTRACT

Targeted amplicon sequencing to identify pathogens, resistance-conferring mutations, and strain types is an important tool in diagnosing and treating infections. However, due to the short read limitations of Illumina sequencing, many applications require the splitting of limited clinical samples between two reactions. Here, we outline single-tube hairpin Illumina sequencing PCR (*his*PCR) which allows for the generation of overlapping amplicons containing Illumina indexes and adapters in a single tube, effectively extending the Illumina read length while maintaining reagent and sample input requirements.

ATTACHMENTS

hisPCR.png

Protocol Info: Jason D Limberis, alina.levin, Joel Ernst, john.metcalfe . hisPCR: A simple, single-tube overlapping amplicon-targeted Illumina sequencing assay. **protocols.io** https://protocols.io/view/hispcr-a-simple-single-tube-overlapping-amplicon-t-cpgfvjtn

Created: Feb 15, 2023

Last Modified: Feb 16, 2023

PROTOCOL integer ID: 77031

Required

Q5 Hot Start High-Fidelity DNA Polymerase - 500 units **New England Biolabs Catalog #M0493L**

dNTPs **Contributed by users**

Agencourt AMPure XP **Beckman Coulter Catalog #A63880**

PowerUp SYBR Green Master Mix **Contributed by users Catalog #A25741**

A thermocycler and a qPCR machine
A magnetic rack

Primers

A	B	C
Primer Set	Direction	Sequence
Rv0678	F1	ACACTCTTTCCCTACACGACGCTCTTCCGATCTtcgatccgctgtggcttggc
Rv0678	F2	GACTGGAGTTCAGACGTGTGCTCTTCCGATCTgccgctcgggatcacacacc
Rv0678	R1	GACTGGAGTTCAGACGTGTGCTCTTCCGATCTttgactcggttggcgggtcg
Rv0678	R2	ACACTCTTTCCCTACACGACGCTCTTCCGATCTcgacgagcgcctcgttgttg
Illumina adapter primer	F	AATGATACGGCGACCACCGAGATCTACAC[i5]ACACTCTTTCCCTACACGACGCTCTTCCGATCT
Illumina adapter primer	R	GATCGGAAGAGCACACGTCTGAACTCCAGTCAC[i7]ATCTCGTATGCCGTCTTCTGCTTG
Illumina_quant_Inner	F	ACACTCTTTCCCTACACGACGCTCTTCCGATCT
Illumina_quant_Inner	R	GACTGGAGTTCAGACGTGTGCTCTTCCGATCT
Illumina_quant_Outer	F	CAAGCAGAAGACGGCATACGAGAT
Illumina_quant_Outer	R	AATGATACGGCGACCACCGAGATC

*e.g., [i5] = A501 = TGAACCTT; [i7] = A701 = ATCACGAC
Lowercase bases are gene specific regions, change these if designing your own primer set

Stage 1 PCR

1

A	B
COMPONENT	Volume (µl)
5X Q5 Reaction Buffer	10
5X Q5 High GC Buffer	10
10 mM dNTPs	1
Q5 High-Fidelity DNA Polymerase	0.5
10µM Forward primer 1	1
10µM Reverse primer 2	1
20 mg/ml BSA	5
Template DNA (~1ng/ul)	5
Nuclease-Free Water	18.5

The total volume is 50ul at this stage

A	B	C	D
Step	Temp (C)	Time (s)	Cycles
Denaturation	98	120	1
Denaturation	98	10	10
Annealing	64	15	
Extension	72	45	
Extension	4	Forever	1

Cycle parameters

As soon as the sample hits 4 °C, proceed to step 2.

Stage 2 PCR

2 Add the below into the reaction while at 4 °C and proceed to the second PCR cycling

A	B
COMPONENT	Volume (µl)
10µM Forward primer 2	0.15
10µM Reverse primer 1	0.15

A	B
10µM Forward Illumina adapter primer	0.35
10µM Reverse Illumina adapter primer	0.35

The total volume is 51ul at this stage

A	B	C	D
Step	Temp (C)	Time (s)	Cycles
Denaturation	98	10	1
Denaturation	98	10	20
Annealing	65	15	
Extension	72	30	
Extension	72	120	1

Cycle parameters

Bead cleanup

3 Add 40 µL of resuspended AMPure XP beads to each reaction in a PCR tube 10m 30s

Mix by pipetting 10x

Incubate 00:05:00 at Room temperature

Place on magnet

Wash 2x with 200 µL freshly-prepared [M] 70 % (v/v) ethanol

Air dry for 00:00:30, don't allow the beads to become cracked

Remove the tubes from the magnetic rack

Add 50 µL 10 mM Tris-HCl pH 8.0 with 50 mM NaCl

NOTE: The BSA in the reaction causes the beads to clump.

Flick the tubes to partially resuspend the beads

Mix by pipetting 10x

Incubate 00:05:00 at Room temperature

Place on the magnet, aspirate 50 µL of the eluant into a new tube

Run 10 µL on a 0.8% agarose gel

Optional: Determine the proportion of amplicons with both ...

1m 50s

4 Dilute the cleaned up amplicons 1:100.
Make the master mix below using Illumina_quant_Inner primer set and a second master mix for Illumina_quant_Outer primer set.

A	B
COMPONENT	Volume (µl)
PowerUp SYBR Green Master Mix (2X)	5
10µM Forward primer	0.5
10µM Reverse primer	0.5
Diluted amplicon	2
Nuclease-Free Water	2

qPCR master mix

Aliquot 8 µL to each well and add in 2 µL of the diluted amplicon.

5

A	B	C	D	E
Step	Temp (C)	Time (s)	Cycles	Ramp Rate (C/s)
UDG activation	50	120	1	2.73
Denaturation	95	120		2.73
Denaturation	95	1	1	2.73
Amplification	60	30		2.11
Capture	60	0	–	
Melt Curve	95	1	1	2.73
	60	20	1	2.11
	95	–	1	0.15
	Capture	–	1	–

Cycle parameters for QuantStudio 3

6 The ratio of the Illumina _quant_Inner primer set to the Illumina _quant_Outer primer set will give the proportion of the DNA in the tube that contain both Illumina adapters and is sequencable (use this for pooling). While the melt curve will provide information on the proportion of each amplicon in the sample.

Expected Result

7

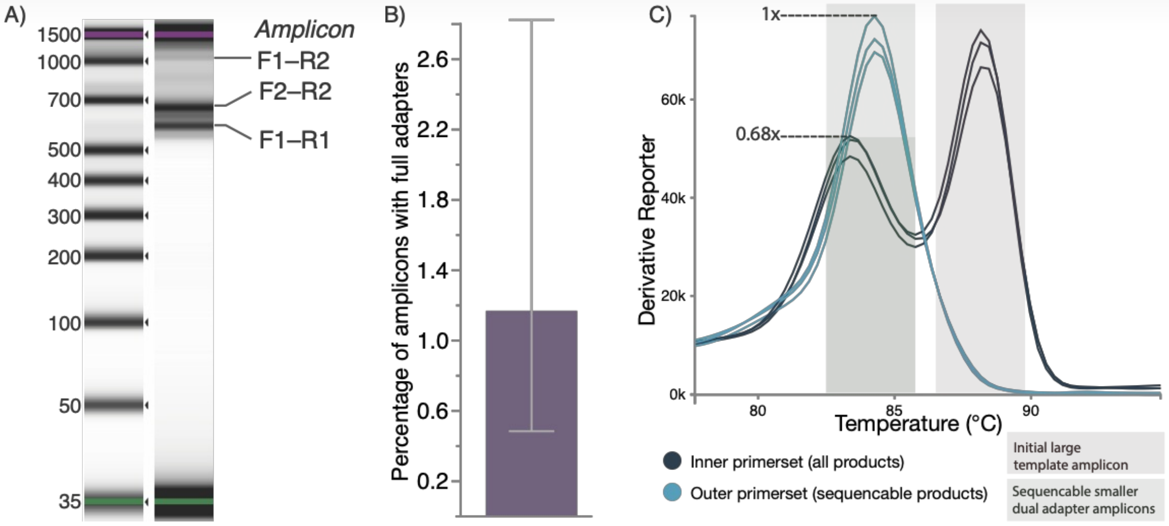

A) hisPCR generated amplicons using two forward and two reverse overlapping primers in a single tube. Amplicons contain either no Illumina adapter, one Illumina adapter, or two Illumina adapters per amplicon. B) The relative proportion of amplicons in the sample containing both Illumina adapters. C) Melt curves showing the expected amplicons for each of the primer sets, and the ratio of amplicons containing both Illumina adapters.
