# Supplementary material for: A simple, single-tube overlapping amplicon-targeted Illumina sequencing assay
Source: PLoS One. 2023 Sep 14;18(9):e0288687. doi: 10.1371/journal.pone.0288687 (PMC10501585; doi:10.1371/journal.pone.0288687)
Supplement: S1 Raw images — (PDF) [file pone.0288687.s004.pdf]

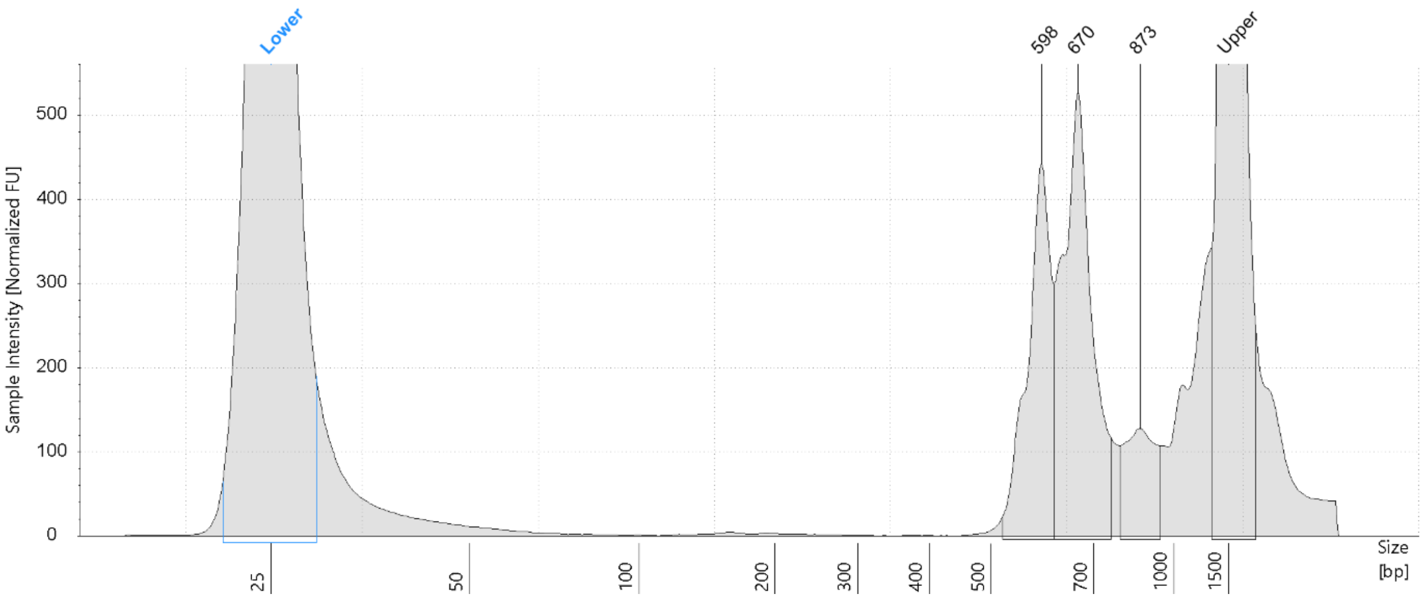

Lower
598
670
873
Upper
500
400
300
200
100
0
Sample Intensity [Normalized FU]
25
50
100
200
300
400
500
700
1000
1500
Size
[bp]

S1_raw_images. Electropherogram output from D1000 Agilent TapeStation of *hiss*PCR generated amplicons using two forward and two reverse overlapping primers in a single tube as shown in Figure 1 A.
